# Occurrence of anterior uveitis in patients with spondyloarthritis treated with tumor necrosis factor inhibitors: comparing the soluble receptor to monoclonal antibodies in a large observational cohort

**DOI:** 10.1186/s13075-020-02187-y

**Published:** 2020-04-26

**Authors:** Gisèle Khoury, Jacques Morel, Bernard Combe, Cédric Lukas

**Affiliations:** Rheumatology Department, Lapeyronie Hospital, 34295 Montpellier cx 5, France

**Keywords:** Spondyloarthritis, Psoriatic arthritis, Uveitis, TNF inhibitors

## Abstract

**Background:**

The objective of this study was to compare in real life the occurrence of anterior uveitis in patients with spondyloarthritis (SpA), including psoriatic arthritis (PsA), treated with the soluble-receptor etanercept (ETA) or monoclonal antibodies (mAbs).

**Methods:**

This was an observational, retrolective study. Patients with SpA who were prescribed anti-TNF agents between 2000 and 2014 were included. The risk of uveitis was interpreted qualitatively (number of subjects with at least one uveitis) and quantitatively (number of uveitis flares for each individual). Models were adjusted for propensity score of receiving preferentially mAbs or ETA.

**Results:**

Four hundred twenty-nine patients were included (302 with SpA and 127 with PsA); 203 received a mAb and 226 ETA as a first TNF-α inhibitor. Probability of uveitis occurring during the first year of treatment was lower with ETA than with mAbs but not significantly (odds ratio 0.94 [95% confidence interval 0.35; 2.54], *p* = 0.90, on qualitative analysis and relative risk 0.62 [0.26; 1.46], *p* = 0.27, on quantitative analysis) after adjustment for the propensity score. The over-time risk of uveitis was numerically higher with ETA than with mAbs, but the differences were not statistically significant.

**Conclusion:**

In this observational study, the risk of uveitis in patients with SpA does not appear to be greater with ETA than with mAb treatment. The occurrence of uveitis in patients receiving an anti-TNF-α agent seems linked more to the history of uveitis than the prescribed molecule.

## Background

Acute anterior uveitis is the most frequent extra-articular manifestation in spondyloarthritis (SpA) and sometimes the first symptom leading to the diagnosis of the disease: 25 to 40% of SpA patients will experience at least one acute anterior uveitis episode during the disease [1, 2]. Although uveitis occurring during SpA usually has good prognosis and responds to local anti-inflammatory treatments [3], it impairs the quality of life of patients and can, mostly when recurrent, result in visual sequelae [4–6].

The incidence of acute anterior uveitis in patients with ankylosing spondylitis was found significantly reduced with infliximab or etanercept (ETA) as compared with placebo [1]. A review of 8 randomized trials showed a decrease in the incidence of uveitis in patients receiving ETA versus placebo [7]. In patients receiving adalimumab, the reduction in incidence as compared with placebo was estimated at 51% [8]. Golimumab has been found effective in uveitis resistant to local treatments, general first-line treatments, and in some cases, other TNF-α inhibitors (TNFi) [9]. Data provided by the randomized study RAPID-Ax-Spa, evaluating uveitis in both ankylosing spondylitis (AS) and non-radiographic axial SpA, also showed a marked decrease in the incidence of acute anterior uveitis with certolizumab versus placebo [10].

Data on ETA are more discordant. The study of the Swedish register of biologics in AS patients showed a significantly higher incidence of uveitis with ETA than with infliximab or adalimumab. This applied to the recurrence of uveitis but also de novo uveitis. Furthermore, patients with a history of uveitis in the 2 years before the introduction of an TNFi showed an increase in the incidence of uveitis during the first 2 years of treatment if ETA was used as the first-line TNFi, whereas the use of infliximab or adalimumab as first-line treatment decreased the incidence [11]. Similarly, another study involving a US database of SpA patients receiving a first TNFi showed a 1.9-fold higher risk of uveitis in the first year with ETA as compared with adalimumab [12], with no significant difference between ETA and infliximab [1, 12]. According to current recommendations, symptoms of SPA (axial, peripheral) as well as extra-articular manifestations must be considered when planning the therapeutic strategy [13].

The main objective of this study was to compare the occurrence of anterior uveitis with ETA and monoclonal antibody (mAb) treatment in patients with SpA during the first year of TNFi treatment in daily practice. Secondary objectives were to compare the occurrence of anterior uveitis with ETA and monoclonal antibody (mAb), prescribed (1) as the first-line TNFi then (2) at any therapeutic lines.

## Methods

### Study design and setting

This observational retrolective study was conducted with a monocentric cohort in the Department of Rheumatology of the university hospital of Montpellier, France. The data were collected from the electronic medical records of adult patients with a clinical diagnosis of axial or peripheral SpA, including psoriatic arthritis (PsA), in whom TNFi agents were initiated. Patients had to fulfill the Assessment of SpondyloArthritis international Society criteria (ASAS) [14] for SpA and CASPAR Classification Criteria for Psoriatic Arthritis for PsA to be included [15]. Data were collected on the date of diagnosis, comorbidities, general treatments and conventional synthetic disease-modifying anti-rheumatic drugs potentially associated with TNFi, the TNFi treatment used and the time elapsed since its introduction, and the uveitis episodes that occurred during pre-treatment and during treatment periods, especially during the first year.

Patients with a short follow-up after the introduction of TNFi therapy (< 2 years) and/or significant time intervals when they were not seen (> 3 years) were considered at risk of missing data. They or their rheumatologist were contacted by phone to obtain information. Patients were not involved in the design, or conduct, or reporting, or dissemination plans of our research.

### Outcome and main analyses

**Fig. 1:**
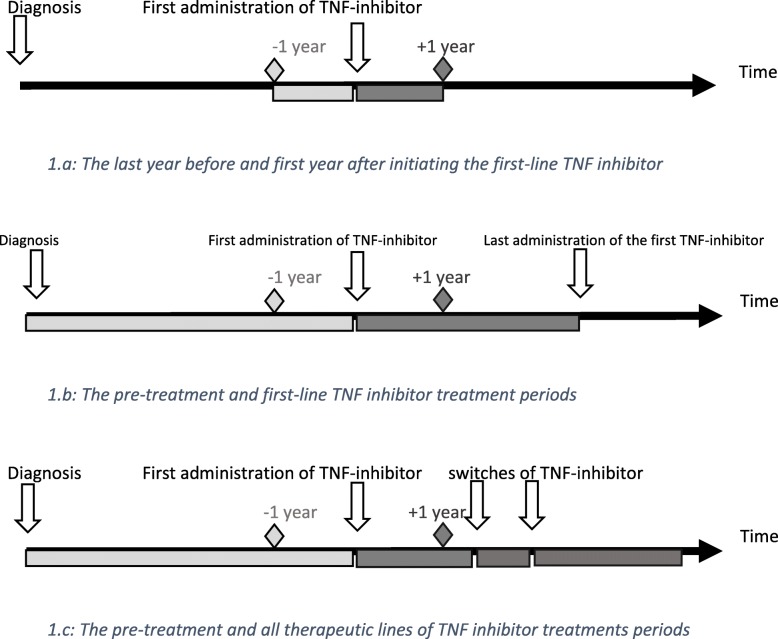
The time intervals involved in the statistical analyses. **a** The last year before and first year after initiating the first-line TNF inhibitor. **b** The pre-treatment and first-line TNF inhibitor treatment periods. **c** The pre-treatment and all therapeutic lines of TNF inhibitor treatment periods

The main outcome was defined in two ways: first by taking into account the occurrence of at least one anterior unilateral uveitis, always confirmed by an ophthalmologist, during the treatment, resulting in a binary variable for qualitative analyses, then by counting the number of uveitis flares during treatment, resulting in a continuous variable for quantitative analyses. Our analyses focused on 3 periods: (1) the first year of TNFi treatment for every patient (Fig. 1a), (2) the whole period when patients received their first TNFi (Fig. 1b), and (3) the period when patients were on a TNFi regardless of the therapeutic line (Fig. 1c).

#### Statistical analysis

Baseline data are presented as number (%) or mean (SD) and were compared by the chi-square test or Student *t* test. To compare the occurrence of uveitis before and after treatment, the MacNemar chi-square test for matched series (each patient being their own control) was used for qualitative analyses. For quantitative analyses, the incidence of uveitis before and during treatment was expressed as uveitis/patient-months (or uveitis/patient-years), and the two incidences were compared by matched Wilcoxon test.

Because patients were excluded from the previous analysis when the number of uveitis flares was unknown, a sensitivity analysis was considered: these patients were assigned a value of 1 for the total number of uveitis events before the introduction of treatment (hypothesis resulting in the most limited estimation of the benefit of the TNFi). The efficacy of ETA and mAbs was compared qualitatively by logistic regression and quantitatively with Poisson regression, estimating odds ratios (ORs) and relative risks (RRs), respectively, with 95% confidence intervals (CIs). Mixed models were used for the analyses that included all therapeutic lines.

Because of the observational design of this study and the channeling bias (confounding by indication) related to the choice of TNFi according to the presence or absence of a known history of one or many uveitis flares, the statistical models used were adjusted for a propensity score. This propensity score aimed at balancing the distribution of confounding factors among baseline characteristics across the ETA- and mAb-treated groups, to approximate the effect of a randomization [16, 17]. Propensity score analysis assigns to every patient a probability between 0 and 1 of receiving ETA as TNFi treatment, according to the patient’s characteristics at the time of the therapeutic decision. The propensity score was defined by a logistic regression model, with the chosen treatment (ETA or mAb) as the dependent variable and the baseline characteristics showing a potential statistical association on univariate analysis (*p* < 0.20) with the choice of TNFi or onset of at least one uveitis in the first year of treatment as independent variables.

## Results

### Patient characteristics (Table 1)

A total of 429 patients were included: 203 received a mAb and 226 ETA (supplementary figure S1). At baseline, extra-articular symptoms, especially inflammatory bowel disease, were less frequent with ETA than with mAb treatment. Other baseline characteristics did not differ between the two groups.

**Table 1:** Baseline characteristics

	Monoclonal antibodies	Soluble receptor (etanercept)	Total
Number of patients	203/429 (47.3)	226/429 (52.7)	429/429
SpA (excluding PsA)	146/203 (71.9)	156/226 (69.0)	302/429 (70.4)
PsA	57/203 (28.1)	70/226 (31.0)	127/429 (29.6)
Men	128/203 (63.1)	133/226 (58.8)	261/429 (60.8)
Age, years, mean (SD)	42.4 (12.3)	44.0 (12.9)	43.2 (12.4)
HLA B27	111/175 (63.4)	119/182 (65.3)	230/357 (64.4)
MD	28/203 (13.8)	44/226 (19.5)	72/429 (16.8)
Time between diagnosis and introduction of the first TNF inhibitor, years, mean (SD)	6.9 (8.2)	7.6 (8.7)	*7.3 (8.5)*
MD	8/203 (3.9)	12/226 (5.3)	*20*/429 (4.7)
History of extra-articular symptoms before treatment	137/197 (69.5)*	*119*/217 (54.8)*	*256*/414 (61.8)
MD	*6/203 (3.0)*	*9*/226 (4.0)	*15*/429 (3.5)
Uveitis	*41/188 (21.8)*	*33*/214 *(15.4)*	*74*/402 (18.4)
MD	*15/203 (7.4)*	*12*/226 (5.3)	*27*/429 (6.3)
Psoriasis	*72/188 (38.3)*	*81*/210 *(38.6)*	*153*/398 (38.4)
MD	*15/203 (7.4)*	*16*/226 (7.1)	*31*/429 (7.2)
IBD	19/203 (9.4)*	2/226 (0.9)*	21/429 (4.9)
csDMARD treatment			
Patients with sDMARD	*54/130 (41.5)*	*56*/151 *(37.1)*	110/281 (39.1)
Patients without sDMARD	*76/130 (58.5)*	*95*/151 *(62.9)*	171/281 (60.9)
MD	*73/203 (36.0)*	*75*/226 *(33.2)*	148/429 (34.5)
Methotrexate	44/130 (33.6)	44/151 (29.1)	88/281 (31.3)
Leflunomide	2/130 (1.5)	2/151 (1.3)	4/281 (1.4)
Sulfasalazine	8/130 (6.2)	10/151 (6.6)	18/281 (6.4)

Data are *n* (%) unless indicated

*MD* missing data, *SD* standard deviation, *IBD* inflammatory bowel disease, *PsA* psoriatic arthritis, *sDMARD* synthetic disease-modifying anti-rheumatic drug

*p < 0.05

### Uveitis occurrence

#### Qualitative description

In total, 73 (6.3%) patients had a history of uveitis, 27 during the year before TNFi start; 19 of 396 (4.4%) patients with available data had at least one uveitis episode during the first year of treatment. With all TNFi lines, 52 (12.9%) patients had at least one uveitis episode (Table 2).

**Table 2:** Occurrence of uveitis during the different time intervals of the study

	Patients with at least one uveitis (qualitative analysis)		Total number of uveitis cases (quantitative analysis)	
**Between diagnosis and start of the first TNF inhibitor**	73		*117*	
**Last year before TNF inhibitor**	*27*		*28*	
**First year of TNF inhibitor**	*ETA*	*9*	*ETA*	*15*
	*mAb*	*10*	*mAb*	*28*
	*Total*	*19*	*Total*	*43*
**First TNF inhibitor**	*ETA*	*23*	*ETA*	*50*
	*mAb*	*19*	*mAb*	*67*
	*Total*	*42*	*Total*	*117*
**All TNF inhibitor lines**	*ETA*	*48*	*ETA*	*62*
	*mAb*	*31*	*mAb*	*108*
	*Total*	*52*	*Total*	*170*

*ETA* etanercept, *mAb* monoclonal antibodies

#### Quantitative description

During the first year of TNFi treatment, 43 uveitis episodes were reported. With all TNFi lines, 170 uveitis episodes were reported; 117 occurred with the first TNFi agent (Table 2).

### Propensity score allocation

The variables selected for the propensity score were occurrence of at least one uveitis in the whole period preceding the introduction of the first TNFi, age, sex, history of inflammatory bowel disease, delay between the diagnosis date and introduction of the first TNFi, and diagnosis of rheumatism (PsA or SpA) (supplementary table S1). This score balanced the population characteristics and probability of being assigned ETA as the first-line TNFi in both groups of patients (Supplementary figure S2, Supplementary figure S3). Despite imperfect performances, this propensity score mainly affected and balanced the two variables that were the most strongly associated with the type of TNFi prescribed and/or to the occurrence of uveitis during the first year of treatment, namely “history of IBD” and “history of uveitis.”

### Qualitative analysis comparing uveitis incidence with ETA versus mAbs

The crude incidence of at least one uveitis episode within 1 year after starting the first TNFi was numerically lower for patients with ETA than with mAbs but not significantly (OR = 0.81 [0.32, 2.03], *p* = 0.65) and was still lower on adjustment for the propensity score but not significantly (OR = 0.94 [0.35, 2.54], *p* = 0.90) (Table 3).

**Table 3:** Incidence of uveitis with mAb and ETA, without and with adjustment for propensity score (PS)

		ETA versus mAb (reference = mAb)			
		Qualitative analysis		Quantitative analysis	
		OR [95% CI]	*p*	RR [95% CI]	*p*
First year of TNF inhibitor treatment	Unadjusted for PS	0.81 [0.32, 2.03]	0.65	0.54 [0.24, 1.24]	0.15
	Adjusted for PS	0.94 [0.35, 2.54]	0.90	0.62 [0.26, 1.46]	0.27
First-line TNF inhibitor	Unadjusted for PS*	1.39 [0.71, 2.72]	0.30	0.97 [0.66, 1.43]	0.89
	Adjusted for PS*	1.89 [0.90, 4.01]	0.09	1.10 [0.70, 1.72]	0.68
All therapeutic lines of TNF inhibitors	Unadjusted for PS*	1.57 [0.71, 3.46]	0.27	1.01 [0.61; 1.69]	0.96
	Adjusted for PS*	1.98 [0.90, 4.37]	0.08	1.21 [0.68, 2.16]	0.53

*OR* odds ratio, *RR* relative risk, *95% CI* 95% confidence interval

*Adjusted for duration of TNF inhibitor treatment

During the first-line TNFi treatment (Fig. 1b), the probability of at least one uveitis episode was higher with ETA than with mAbs but not significantly (OR = 1.39 [0.71, 2.72], *p* = 0.3) and was higher on adjustment for the propensity score but still not significantly (OR = 1.89 [0.90, 4.01], *p* = 0.09) (Table 3).

When considering all prescribed therapeutic lines of TNFi agents, the probability of at least one uveitis episode was numerically higher with ETA than with mAbs but not significantly (OR = 1.57 [0.71, 3.46], *p* = 0.27) and was further increased on adjustment for the propensity score but still not significantly (OR = 1.98 [0.90, 4.37], *p* = 0.08) (Table 3).

### Quantitative analysis comparing uveitis incidence with ETA versus mAbs

On quantitative analysis, the risk of uveitis within 1 year after starting the first TNFi was lower with ETA than with mAbs but not significantly (RR = 0.54 [0.24; 1.24], *p* = 0.15) and was still lower on adjustment for the propensity score but not significantly (RR = 0.62 [0.26, 1.46], *p* = 0.27) (Table 3).

When considering the first-line TNFi treatment, the risk of uveitis was reduced with ETA versus mAbs but not significantly (RR = 0.97 [0.66, 1.43], *p* = 0.89) and was increased on adjustment for the propensity score but not significantly (RR = 1.10 [0.70; 1.72], *p* = 0.68) (Table 3).

When considering total TNFi treatment exposure (all prescribed therapeutic lines), risk of uveitis was higher with ETA than with mAbs but not significantly (RR = 1.01 [0.61; 1.69], *p* = 0.96) and was further increased on adjustment for the propensity score but still not significantly (RR = 1.21 [0.68, 2.16], *p* = 0.53) (Table 3).

### Uveitis occurrence before and during TNFi treatment

We found no significant decrease in the number of participants with at least one uveitis episode when comparing the incidence of uveitis between the year before starting treatment and the first year of the first-line TNFi (*p* = 0.17) (Fig. 1a, Table 4). When comparing the incidence of uveitis over the previous year and after the TNFi start, the main analysis did not find any significant difference between the two groups (0.0066 uveitis/patient-months in the previous year and 0.011 uveitis/patient-months in the first TNFi year, *p* = 0.80; Table 5). Also, the sensitivity analysis (number of uveitis episodes set to 1) did not reveal a significant difference between the two incidences (*p* = 0.69; Table 5).

**Table 4:** Qualitative analysis of the occurrence of at least 1 uveitis before and during the first year of TNF inhibitor treatment in patients with SpA or PsA

		Uveitis ≥ 1 during the first year of TNF inhibitor		
		Yes	No	*p*
Uveitis ≥ 1 in the year before TNF inhibitor	Yes	10	17	0.17
	No	9	325

**Table 5:** Quantitative analysis of the incidence of uveitis in the last year before and during the first year of TNF inhibitor treatment in patients with SpA or PsA

		Main analysis *n* = 350	Sensitivity analysis *n* = 351
In the last year before TNF inhibitor	Uveitis/patient-months, mean (SD)	0.0066 (0.0333)	0.0066 (0.0333)
	Uveitis/100 patient-years	7.92	7.92
In the first year of TNF inhibitor	Uveitis/patient-months, mean (SD)	0.011 (0.080)	0.011 (0.080)
	Uveitis/100 patient-years	13.2	13.2
*p* value		0.80	0.69

We compared the incidence of uveitis with first-line TNFi treatment versus the incidence during the time between the diagnosis of SpA and the introduction of the first TNFi (Fig. 1b). The qualitative analysis showed a significant decrease in the number of patients with at least one uveitis episode (*p* = 0.0002) (supplementary table S2). The quantitative analysis as well as the sensitivity analysis revealed no significant difference in pre-treatment and treatment incidences (*p* = 0.19 and *p* = 0.16, respectively) (Supplementary table S3).

Finally, we compared the incidence of uveitis between the total period under TNF treatment, considering all therapeutic lines, and between the date of diagnosis and introduction of the first TNFi (Fig. 1c). The qualitative analysis revealed a significant decrease in incidence under treatment (*p* = 0.049) (supplementary table S2), whereas the quantitative analysis revealed a significant increase in incidence under treatment (*p* = 0.04 for the main analysis and *p* = 0.03 for the sensitivity analysis; supplementary table S3).

## Discussion

According to the main result of our study, the type of TNFi chosen does not affect the risk of uveitis in the year after the initiation of TNFi treatment for SpA, this interpretation remaining subject to the limitations of our study. The comparisons, both qualitative and quantitative analyses of the incidence of uveitis in the year after introducing the TNFi, found a non-significant reduced risk of developing uveitis with soluble-receptor ETA versus mAb treatment. Adjustment for the propensity score tended to reduce the difference that might have favored ETA, which was related rather to differences in baseline covariates according to the treatment assigned. Limiting a prescription for ETA in patients with a history of uveitis might erroneously result in an apparently protective effect of ETA.

The lack of significant difference in the occurrence of uveitis ETA and mAb contrasts with what was demonstrated in two major observational studies, despite very similar conditions to our cohort [11, 12]. In these two studies, uveitis incidence was higher with ETA than with adalimumab or infliximab over 1 or 2 years of treatment with TNFi. These studies did not use propensity score adjustment, and patients with a history of uveitis were excluded from Wendling et al. study [12]. In our qualitative analysis, the risk of at least one uveitis episode with first-line TNFi and all therapeutic lines was higher with ETA than with mAbs but not significantly. The magnitude of the increased risk with ETA (almost twice that for mAbs) was similar to that found in the Swedish Rheumatology Quality Register; the risk of uveitis at 2 years was increased by 1.99-fold with ETA versus infliximab [11]. In our quantitative analysis, the risk of uveitis did not differ with ETA and mAbs. This result is consistent with studies using quantitative analysis, such as the Braun et al. study, which did not reveal any difference between ETA and infliximab [1].

Our study also suggested that the risk of uveitis is not associated with the treatment used but is associated with the history of the disease and disease activity. This finding is particularly true because the prevalence of uveitis was, according to our study, the main risk factor for uveitis incidence with TNFi treatment (*p* < 0.001) (Table 5). This increased incidence during the first year of treatment should be noted. The introduction of the first anti-TNF agent in PsA and SpA is usually a result of clinical worsening of disease activity, resulting in a high expected incidence of uveitis in the first months after treatment start, as suggested by the higher proportion of de novo uveitis compared to recurrent uveitis on qualitative analysis in the first year of treatment (4.71% and 2.77%, respectively).

The reduction in the incidence of uveitis when all therapeutic lines were considered in comparison to the incidence in patients receiving first-line TNFi treatment reveals that most events occur during the first year of treatment. This reinforces our choice to define the main objective of this study with the first year after the introduction of the first TNFi. This is also the period when the risk of missing data is lowest because patients are monitored regularly.

Our study has several limitations. First, it reflects the practices and prescribing habits of a single rheumatology department. The low number of events may also have resulted in a lack of power. The data collection in this retrolective study may also have resulted in potential missing data in medical records and therefore lower prevalence and incidence of uveitis, especially in the pre-treatment period, than values usually reported in the literature [1, 18–20]. A monitoring bias may have contributed to an increase in the number of events reported during treatment because patients were more frequently reviewed and questioned when treated. Recovery of missing data by phone calls was hampered by a large number of patients who could not be reached and substantial memory bias in those contacted. Finally, the observational design of the study implied biases related to the prescription of TNFi, such as a channeling bias.

The main strength of this study is that data were collected from current clinical practice. Almost all patients with SpA or PsA who received TNFi agents in our department were recruited. Given that every first prescription of a TNFi in our country can only be given during a hospital visit, our population is highly representative of the target population. Channeling bias was limited by the use of the propensity score. Indeed, it reduced the confounding biases associated with a different distribution of characteristics measured at baseline and results in pseudo-randomization by rebalancing the confounding factors in the compared treatment groups.

Although the mechanisms of action of the soluble-receptor ETA and mAbs are different [21], our results did not allow us to conclude that ETA or mAbs were more effective against the occurrence of uveitis in the first year of TNFi treatment. When considering a longer follow-up period, the risk of uveitis appeared potentially greater in patients receiving ETA than mAbs, although not significantly.

## Conclusion

These results, in addition to those already published on the same topic, suggest that the choice of TNFi should consider the history of extra-articular symptoms, because the occurrence of uveitis in patients receiving a TNFi seems to be linked more to the patient’s history of uveitis than the prescribed molecule, while considering the aforementioned limits of this study. Also, a systematic preference for an mAb instead of soluble receptor is not justified in most patients but should be considered in high-risk patients with multiple, frequent, and/or complicated uveitis episodes.

## Supplementary information

**Additional file 1: Supplementary figure 1**: Flowchart of the study.

**Additional file 2: Supplementary table S1**: Statistical associations of patient characteristics at baseline with the first TNF inhibitor choice and with occurrence of at least 1 uveitis during first-line TNF inhibitor treatment.

**Additional file 3: Supplementary figure 2**: Propensity score distribution in the two groups of patients (monoclonal antibodies or soluble receptor at baseline).

**Additional file 4: Supplementary figure S3**: change in standardized mean difference of variables included in the propensity score before and after wheighting.

**Additional file 5: Supplementary table S2**: Qualitative analysis of the occurrence of at least 1 uveitis before and during TNF inhibitor treatment in patients with SpA or PsA.

**Additional file 6: Supplementary table S3**: Quantitative analysis of the incidence of uveitis before and during TNF inhibitor treatment in patients with SpA or PsA.

## Data Availability

The datasets used and/or analyzed during the current study are available from the corresponding author on reasonable request.
