# Supplementary figures and images for: Occurrence of anterior uveitis in patients with spondyloarthritis treated with tumor necrosis factor inhibitors: comparing the soluble receptor to monoclonal antibodies in a large observational cohort
Source: Arthritis Res Ther. 2020 Apr 26;22:94. doi: 10.1186/s13075-020-02187-y (PMC7184699; doi:10.1186/s13075-020-02187-y)

Supplementary figure 1: Flowchart of the study

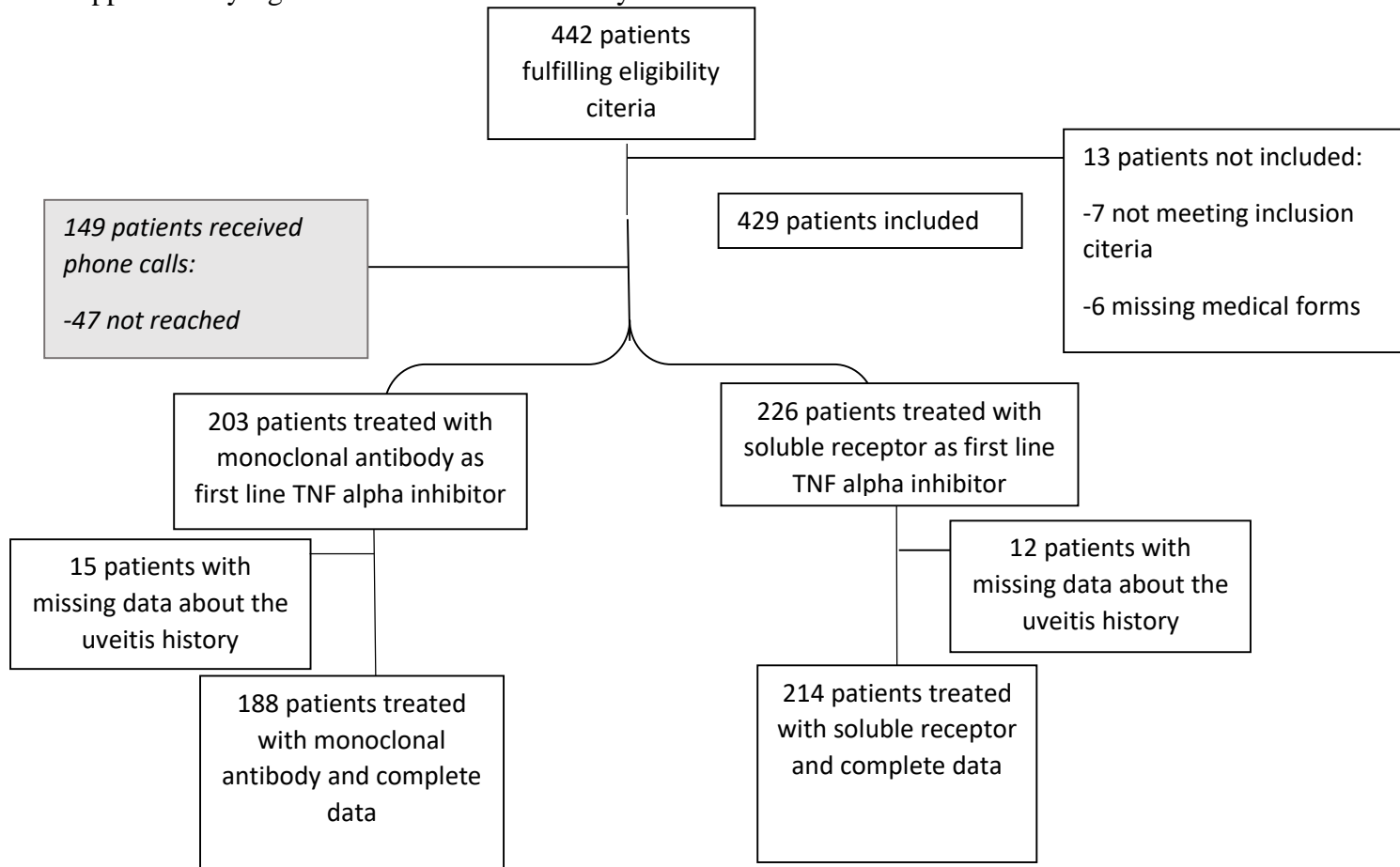

Supplement: Supplementary file 1 — Additional file 1: Supplementary figure 1: Flowchart of the study. [file 13075_2020_2187_MOESM1_ESM.pdf]
