# Supplementary material for: Occurrence of anterior uveitis in patients with spondyloarthritis treated with tumor necrosis factor inhibitors: comparing the soluble receptor to monoclonal antibodies in a large observational cohort
Source: Arthritis Res Ther. 2020 Apr 26;22:94. doi: 10.1186/s13075-020-02187-y (PMC7184699; doi:10.1186/s13075-020-02187-y)
Supplement: Supplementary file 2 — Additional file 2: Supplementary table S1: Statistical associations of patient characteristics at baseline with the first TNF inhibitor choice and with occurrence of at least 1 uveitis during first-line TNF inhibitor treatment. [file 13075_2020_2187_MOESM2_ESM.docx]

Supplementary table S1: Statistical associations of patient characteristics at baseline with the first TNF inhibitor choice and with occurrence of at least 1 uveitis during first-line TNF inhibitor treatment

| Baseline characteristics | p-value of the association with the first TNF inhibitor choice | p-value of the association with occurrence of at least 1 uveitis during first-line TNF inhibitor |
| --- | --- | --- |
| History of uveitis before TNF inhibitor treatment | 0.13 | <0.001 |
| Delay between diagnosis and introduction of the first TNF inhibitor | 0.45 | 0.001 |
| Use of sDMARDs | 0.45 | 0.47 |
| HLAB27 status | 0.7 | 0.99 |
| Age | 0.2 | 0.29 |
| Sex | 0.4 | 0.046 |
| Diagnosis (SpA or PsA) | 0.51 | 0.068 |
| History of IBD | 0.001 | 0.99 |
| History of psoriasis | 0.96 | 0.43 |

*HLAB27 : human leukocyte antigen B27*
