# Supplementary material for: Occurrence of anterior uveitis in patients with spondyloarthritis treated with tumor necrosis factor inhibitors: comparing the soluble receptor to monoclonal antibodies in a large observational cohort
Source: Arthritis Res Ther. 2020 Apr 26;22:94. doi: 10.1186/s13075-020-02187-y (PMC7184699; doi:10.1186/s13075-020-02187-y)

Supplementary figure 2 :  Propensity score distribution in the two groups of patients (monoclonal antibodies or soluble receptor at baseline)

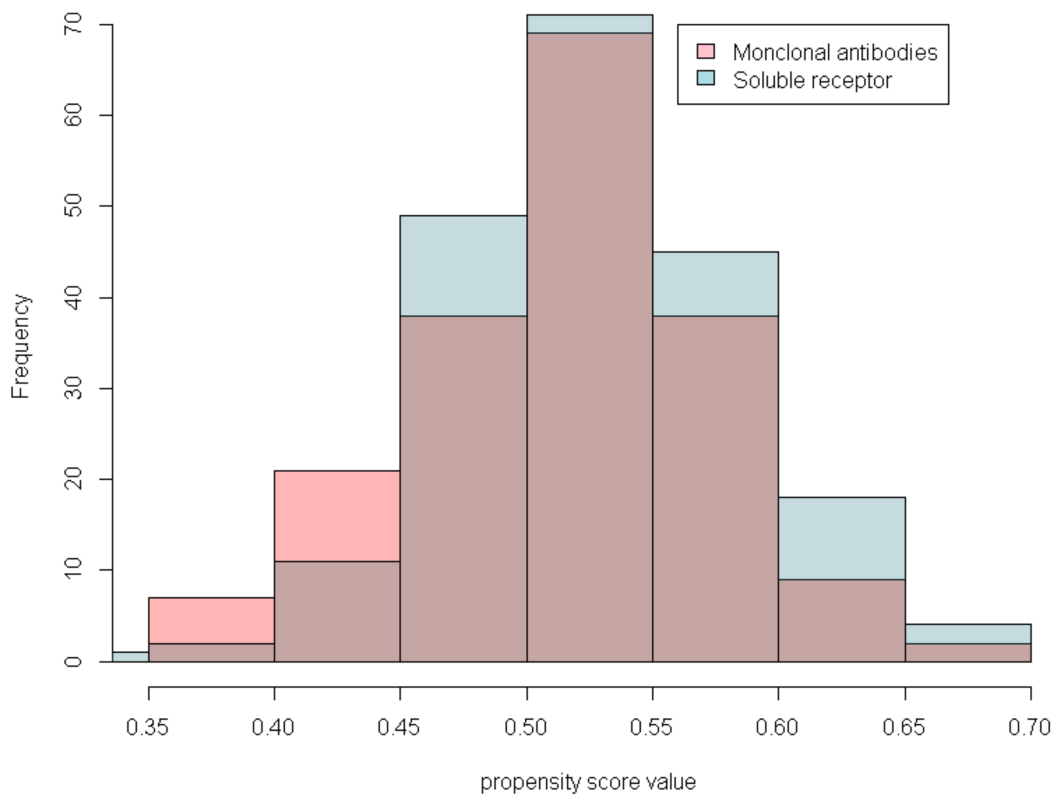

Supplement: Supplementary file 3 — Additional file 3: Supplementary figure 2: Propensity score distribution in the two groups of patients (monoclonal antibodies or soluble receptor at baseline). [file 13075_2020_2187_MOESM3_ESM.pdf]
