# Supplementary material for: Occurrence of anterior uveitis in patients with spondyloarthritis treated with tumor necrosis factor inhibitors: comparing the soluble receptor to monoclonal antibodies in a large observational cohort
Source: Arthritis Res Ther. 2020 Apr 26;22:94. doi: 10.1186/s13075-020-02187-y (PMC7184699; doi:10.1186/s13075-020-02187-y)
Supplement: Supplementary file 4 — Additional file 4: Supplementary figure S3: change in standardized mean difference of variables included in the propensity score before and after wheighting. [file 13075_2020_2187_MOESM4_ESM.pdf]

Supplementary figure S3: change in standardized mean difference of variables included in the propensity score before and after wheighting

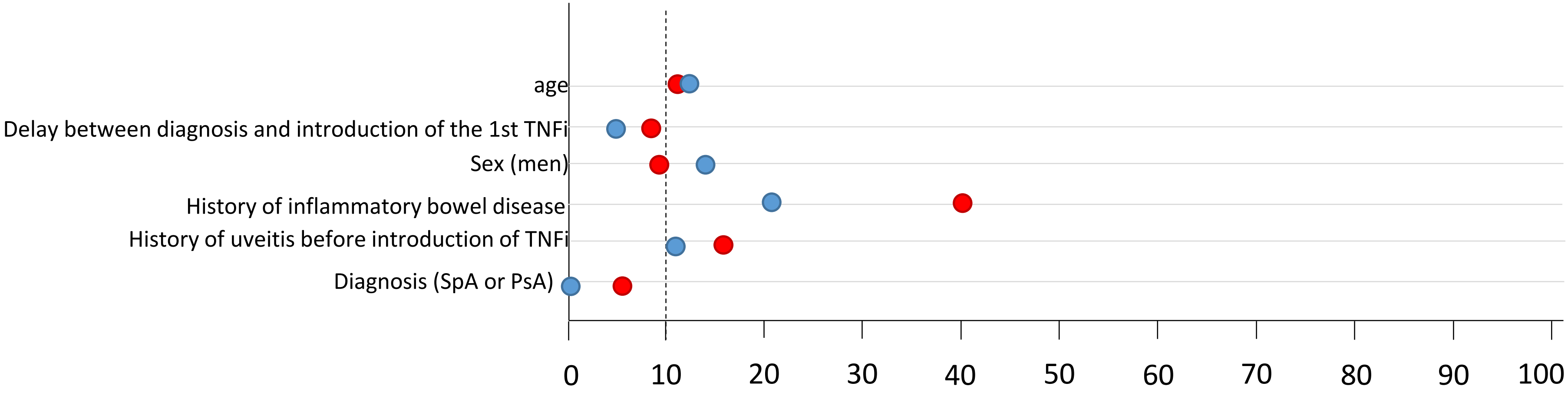

Standardized mean difference before propensity score wheighting

Standardized mean difference after propensity score wheighting

TNFi: TNF alpha inhibitor treatment
SpA: spondyloarthritis
PsA: psoriatic arthritis
