# Supplementary material for: Occurrence of anterior uveitis in patients with spondyloarthritis treated with tumor necrosis factor inhibitors: comparing the soluble receptor to monoclonal antibodies in a large observational cohort
Source: Arthritis Res Ther. 2020 Apr 26;22:94. doi: 10.1186/s13075-020-02187-y (PMC7184699; doi:10.1186/s13075-020-02187-y)
Supplement: Supplementary file 5 — Additional file 5: Supplementary table S2: Qualitative analysis of the occurrence of at least 1 uveitis before and during TNF inhibitor treatment in patients with SpA or PsA. [file 13075_2020_2187_MOESM5_ESM.docx]

|  |  | Uveitis ≥1 during the first TNF inhibitor | | |  |  | Uveitis ≥1 during all TNF inhibitor lines | | |
| --- | --- | --- | --- | --- | --- | --- | --- | --- | --- |
|  |  | Yes | No | p |  |  | Yes | No | p |
| Uveitis ≥1 before TNF inhibitor | Yes | 25 | 48 | 0.0002 | *Uveitis ≥1 before TNF inhibitor* | Yes | 6 | 67 | 0.049 |
|  | No | 17 | 311 |  |  | No | 46 | 282 |  |

Supplementary table S2: Qualitative analysis of the occurrence of at least 1 uveitis before and during TNF inhibitor treatment in patients with SpA or PsA

SpA : spondyloarthritis ; PsA : psoriatic arthritis
