# Supplementary material for: Occurrence of anterior uveitis in patients with spondyloarthritis treated with tumor necrosis factor inhibitors: comparing the soluble receptor to monoclonal antibodies in a large observational cohort
Source: Arthritis Res Ther. 2020 Apr 26;22:94. doi: 10.1186/s13075-020-02187-y (PMC7184699; doi:10.1186/s13075-020-02187-y)
Supplement: Supplementary file 6 — Additional file 6: Supplementary table S3: Quantitative analysis of the incidence of uveitis before and during TNF inhibitor treatment in patients with SpA or PsA. [file 13075_2020_2187_MOESM6_ESM.docx]

Supplementary table S3 : Quantitative analysis of the incidence of uveitis before and during TNF inhibitor treatment in patients with SpA or PsA

| Incidence before TNF inhibitor introduction and during first TNF inhibitor treatment | | | |
| --- | --- | --- | --- |
|  |  | Main analysis  n=334 | Sensitivity analysis*  n=337 |
| Before TNF inhibitor | Uveitis/patient-months, mean (SD) | 0.0040 (0.023) | 0.00420 (0.023) |
|  | Uveitis/100 patient-years | 4.81 | 5.04 |
| With first TNF inhibitor | Uveitis/patient-months (SD) | 0.0086 (0.056) | 0.0086 (0.056) |
|  | Uveitis/100 patient-years | 10.32 | 10.32 |
| p value | | 0.19 | 0.16 |
| Incidence before TNF inhibitor introduction and during all TNF inhibitor lines of treatment | | | |
|  |  | Main analysis  n=334 | Sensitivity analysis*  n=344 |
| Before TNF inhibitor | Uveitis/patient-months, mean (SD) | 0.0040 (0.023) | 0,0042 (0.023) |
|  | Uveitis/100 patient-years | 4.81 | 5.04 |
| With all TNF inhibitor lines | Uveitis/patient-months, mean (SD) | 0.0052 (0.022) | 0.0057 (0.023) |
|  | Uveitis/100 patient-years | 6.24 | 6.84 |
| p | | 0.04 | 0.03 |

SpA : spondyloarthritis ; PsA : psoriatic arthritis

* *Because patients were excluded from the previous analysis when the number of uveitis flares was unknown, a sensitivity analysis was considered: these patients were assigned a value of 1 for the total number of uveitis events before the introduction of treatment*
